# Effects of Climate Change Scenarios on Population and Distribution Pattern of Tree–Ferns in Nepal

**DOI:** 10.1002/ece3.71179

**Published:** 2025-03-30

**Authors:** Ripu M. Kunwar, Dipak Khadka, Khum Thapa‐Magar, Binaya Adhikari, Durga H. Kutal, Rama Ghimire, Komal R. Kafle, Sony Baral, Gokarna J. Thapa, Ananta Bhandari

**Affiliations:** ^1^ Research Centre for Applied Science and Technology Tribhuvan University Kirtipur Nepal; ^2^ South China Agricultural University Guangzhou China; ^3^ Institute of Arctic and Alpine Research University of Colorado Boulder Colorado USA; ^4^ Department of Biology University of Kentucky Lexington Kentucky USA; ^5^ Augusta University St. Augusta Georgia USA; ^6^ Central Department of Environmental Science Tribhuvan University Kirtipur Nepal; ^7^ District Forest Office Kaski Nepal; ^8^ Office of Deans, Institute of Forestry Tribhuvan University Kirtipur Nepal; ^9^ WWF Nepal Program Kathmandu Nepal

**Keywords:** climate change, distribution, mid‐hills, modeling, tree‐ferns

## Abstract

By studying the population structure and spatial characteristics, the relationship between tree‐ferns and the environment can be reflected, which has high practical significance. In this study, we employed an ensemble distribution model to evaluate the relative contribution of various environmental variables and predict suitable habitats for tree‐fern across past, present, and future periods. Fieldwork was carried out between May–June 2019 and September 2022 in 11 districts of Nepal for population sampling and collecting the geocoordinates. Additional geocoordinates were collected from secondary sources such as previous literature, herbarium records, and online resources. We reported the occurrence of tree‐ferns from 28 districts within the altitudinal range of 300–2500 m. Longitudinally, the species is distributed only in central and eastern Nepal, with maximum density in central Nepal's Kaski and Lamjung districts. The central mid‐hills of Koshi and Gandaki provinces, particularly with the moist habitats and maximum rainfall, are suitable for the distribution of tree‐ferns. The projected distribution is influenced mainly by the mean temperature of the coldest quarter—Bio11 (34.9%), precipitation in dry months—Bio14 (34.5%), and mean annual temperature—Bio1 (33.9%). Climate extreme variables (maximum temperature in warmest months—Bio5, minimum temperature in coldest months—Bio6, precipitation in wettest months—Bio13, precipitation in wettest quarter—Bio16) contract the future distribution of species. The result portrays an expansion of suitable habitat for tree‐ferns while minor contractions are predicted in four districts of Bagmati province. As the Gandaki province receives the highest rainfall and the Koshi province has rich soil moisture, and precipitation plays a significant role in distribution, humid riverine places of Koshi and Gandaki support tree‐fern populations. Tree‐ferns could be an indicator species of the moist and humid climate. Given the extensive distribution in Nepal, India, and China, sustainable conservation of tree‐ferns through a species conservation action plan holds broader implications.

## Graphical abstract

Tree‐ferns are distributed only in central and eastern Nepal with maximum density in Kaski and Lamjung districts, Central Nepal. This reveals that the central mid‐hills of Koshi and Gandaki provinces, particularly with the moist habitats and maximum rainfall areas, are suitable for tree‐ferns. The projected distribution is influenced mostly by the mean temperature of the coldest quarter—Bio11 (34.9%), precipitation in dry months—Bio14 (34.5%), and mean annual temperature—Bio1 (33.9%).

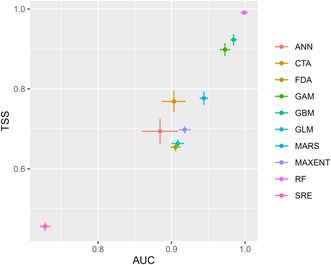

## 1 Introduction

Ferns are an ancient group of vascular plants that evolved during the Carboniferous period (Stampede 2012). Among 12,000 species of ferns, the genus *Alsophila* (tree‐fern) comprises about 322 species (CoL 2024). Scaly ferns, those with thorns in the leaf of *Alsophila*, are a group of primarily tree‐forming ferns with fossil evidence dating back to their origin in the Middle Jurassic age (Tryon and Tryon 1982). Scaly tree‐ferns are thus often informally regarded as living fossils due to their long historical and evolutionary significance (Bystriakova et al. 2010; Rajapaksha et al. 2024). There are five species of *Alsophila* in Nepal: *A. spinulosa* , *A. sollyana*, *A. khasyana* , *A. henryi* , and *A. brunoniana* (Kandel 2020). Among them is *A. spinulosa* (Wall. ex Hook.) R. M. Tyron, common in Nepal and native to Asia (Xie et al. 2023), with a wide range of distribution in China, Japan, Vietnam, Cambodia, Thailand, Myanmar, Bangladesh, Bhutan, Nepal, and India (Chen et al. 2023). This is an arborescent species that can reach heights up to 15 m and commonly grows between 300 and 2200 m above sea level (asl) (Kandel 2020). It is identified by detecting its spiny rachis and young sori surrounded by a thin white indusium (Fraser‐Jenkins et al. 2015). Other species are more or less similar, with or without spiny rachis and different arrangements of sori. Difficulty in identifying the tree‐ferns (Roberts et al. 2005), and limited taxonomic capacity in Nepal (Kunwar et al. 2021) allowed us to group all our samples as tree‐ferns.

Tree‐ferns are an important element, especially in tropical forests, and they contribute variously by providing different ecosystem services such as air, microhabitat for many epiphytic plants, and nesting sites for birds and insects (Jones et al. 2007). Humid, damp, sheltered slopes and moist gullies to hill cloud forests are typical habitats of tree‐ferns (Paul et al. 2015; Mishra and Behera 2020). In the tropical and subtropical regions of South and Southeast Asia, tree‐ferns possess high economic value, being utilized by indigenous groups for food, medicine, and ornaments (Rybczyński and Mikula 2011; Poudel et al. 2018; Kandel 2020; Dhamala et al. 2021; Bhattarai and Kunwar 2022; Rajapaksha et al. 2024).

Due to increasing habitat destructions, deforestation, and over‐exploitation, many valuable plants, including tree‐ferns, have increasingly been threatened (Dhamala et al. 2021). Recent studies have revealed tree‐ferns' rapid demographic decline, including *A. spinulosa* (Bhattarai et al. 2018; Huang et al. 2022; WWF Nepal 2022). Besides anthropogenic pressure, climate change is another factor influencing plant species' distribution (Hussain et al. 2024). All these threats and challenges control tree seedling establishment and sapling survival and may affect the population dynamics and species distribution (Gomez‐Aparicio et al. 2008). The distribution of tree‐ferns is also affected by various other factors such as geology, soil, slope, aspect, moisture, humidity, habitat conditions, and historical accounts (Tuomisto et al. 2003; Merino et al. 2023). Tree‐ferns are restricted in distribution due to their habitat‐specific characteristics (Wei et al. 2021).

Given the prehistoric phylogeny and present exploitation patterns, *A. spinulosa* is listed for conservation (Appendix II by CITES, Threatened category by IUCN 2021) (Dhamala et al. 2021). In China, *A. spinulosa* is included in a National Protection Category on the Red List (Chen et al. 2023). Therefore, assessing the biology, ecology, and diversity of tree‐ferns is imperative in the changing global context. However, little is known about its population and distribution in Nepal. For long‐term management of the impact of climatic and anthropogenic challenges, distribution modeling is an essential tool (Boral and Moktan 2024). The relationship between tree‐ferns and the environment can be drawn by mapping the population structure and spatial characteristics, which have high practical implications for conservation (Budha et al. 2023; Yuan et al. 2023). In this connection, we aimed to determine the present population and distribution of tree‐ferns in Nepal with a detailed account of future distribution in different climate change scenarios in 2050 and 2080 by utilizing the Biomod‐2 ensemble modeling approach.

## 2 Materials and Methods

### 2.1 Study Area and Data Collection

Nepal is divided into three distinct regions, Western (80°E–83°E), Central (83°E–86°30′E), and Eastern (86°30′E–88°12′E) based on floral composition (Banerji 1963; Stearn 1960). Administratively, the country comprises seven provinces, 77 districts, and 753 local bodies, whereas it has five distinct elevational/vertical zones from north to south: (i) High Himalaya (above 5000 m above sea level) with 24% area, (ii) High‐mountains (3000–5000 m asl) with 20% area, (iii) Mid‐hills (1000–3000 m asl) with 30% area, (iv) Siwalik (500–1000 m asl) with 12% area, and (v) Tarai (< 500 m asl) with 14% area (MoSTE 2014) at the physiographic level.

**FIGURE 1:**
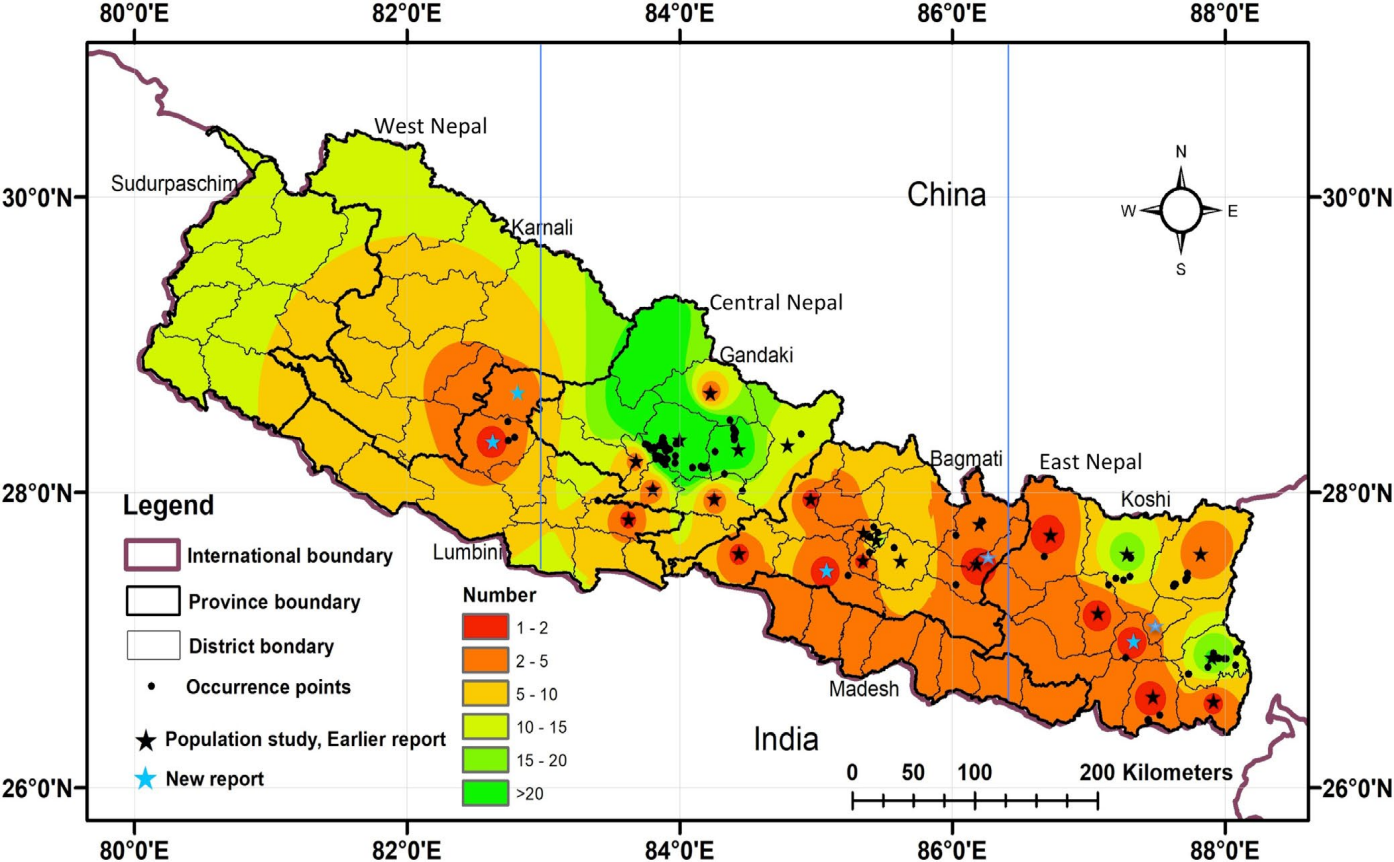
Map showing the distribution of occurrence points (black dots, modeling in color), population study points and earlier reference points of tree‐ferns (star symbol) in Nepal.

We carried out two extensive fieldworks from May to July 2019 and September 2022. We laid 65 quadrats, each measuring 10 m × 10 m in 16 transects (each measuring 200–200 m × 50–100 m) along the trails following convenience sampling and keeping quadrats at least 10 m apart. As tree‐ferns are sparsely distributed in the sites, we laid quadrats where the tree‐ferns were spotted and accessible to carry out a population and georeferencing study. We surveyed a 20‐ha area of 11 districts in 13 locations from Gandaki, Koshi, and Bagmati provinces, collected georeference points (*n* = 134) and assessed the tree‐ferns population. Additional population data and geocoordinate points (*n* = 113) of the species were collected from a review of literature (Bhuju and Joshi 2009; I. Thapa 2017; Bhattarai et al. 2018; Poudel et al. 2018; Dhamala et al. 2021), voucher specimens deposited at National Herbarium and Plant Laboratories (KATH) https://plantdatabase.kath.gov.np/plants/search and Tribhuvan University Central Herbarium (TUCH), and online sources including Flora of Nepal (https://www. floraofnepal.org), iNaturalist (https://www.inaturalist.org/), the Global Biodiversity Information Facility (https://www.gbif.org/), the Royal Botanic Garden at Edinburgh, United Kingdom (RBGE; https://data.rbge.org.uk/search/herbarium/), and the Herbarium at the University of Tokyo, Japan (TI; https://umdb.um.u‐tokyo.ac.jp/DShokubu/). The occurrence points were verified, and the population and conservation of species were recorded through consultation with local communities, including 43 participants. A total of 247 (N) geospatial occurrence points of tree‐ferns from 23 districts were compiled and analyzed for distribution modeling. The association between the variables (population, altitude, latitude, rainfall, and temperature) was tested by applying smooth least squares regression. Participatory maps were used to figure out and locate the trail and species' locations (Figure 1).

### 2.2 GIS Mapping, Distribution Survey and Habitat Modeling

For modeling, we incorporated 5000 pseudo points that were generated within the predicted areas of a polygon. For model calibration, we used 80% of the presence and pseudo‐absence data sets for model development and 20% for the predictive power of each model with three‐fold cross‐validation following the Boyce index (Hirzel et al. 2006). We assessed the model performance using the area under the curve (AUC) and the True Skill Statistic (TSS), sensitivity, and specificity (Allouche et al. 2006). By using the multi‐collinearity test, 22 (19 bioclimatic and three physiographic) variables were reduced to six, which include annual mean temperature (Bio1), temperature seasonality (Bio4), minimum temperature of coldest months (Bio6), mean temperature of coldest quarter (Bio11), annual precipitation (Bio12), and precipitation in driest months (Bio14) by eliminating the cross‐correlated variables. This enhances the accuracy of the prediction modeling. Before the analysis, the data was rarified for 1 km^2^ using the spThin R package (Aiello‐Lammens et al. 2015). Therefore, we had no more than one presence data point at each grid cell (1 km × 1 km) to reduce spatial autocorrelation. All the data processing for the modeling process was conducted using R 4.2 “Biomod2” and “eSDM” packages in R 4.2 (Thuiller et al. 2024; R Development Core Team 2022). We used the default 10 algorithms of eSDM and the consensus model for each species including general additive model (GAM), general linear model (GLM), generalized boosting model (GBM), Random forest (RF), artificial neural network (ANN), classification tree analysis (CTA), flexible discriminant analysis (FDA), multiple adaptive regression splines (MARS), surface range envelope (SRE), and maximum entropy (MAXENT). We chose shared socioeconomic pathways (SSP126, SSP245, SSP370, SSP585) and the present year 2024, 2050, and 2080 for modeling. Modeling output ranged from 0 to 1, where the value close to 1 hinted at a greater probability of occurrence. We categorized the probability pixels by 50% cut‐off. Any pixel higher than or equal to 50% cut‐off value was considered a presence point.

## 3 Results

### 3.1 Model Performances and Key Environmental Variables

**FIGURE 2:**
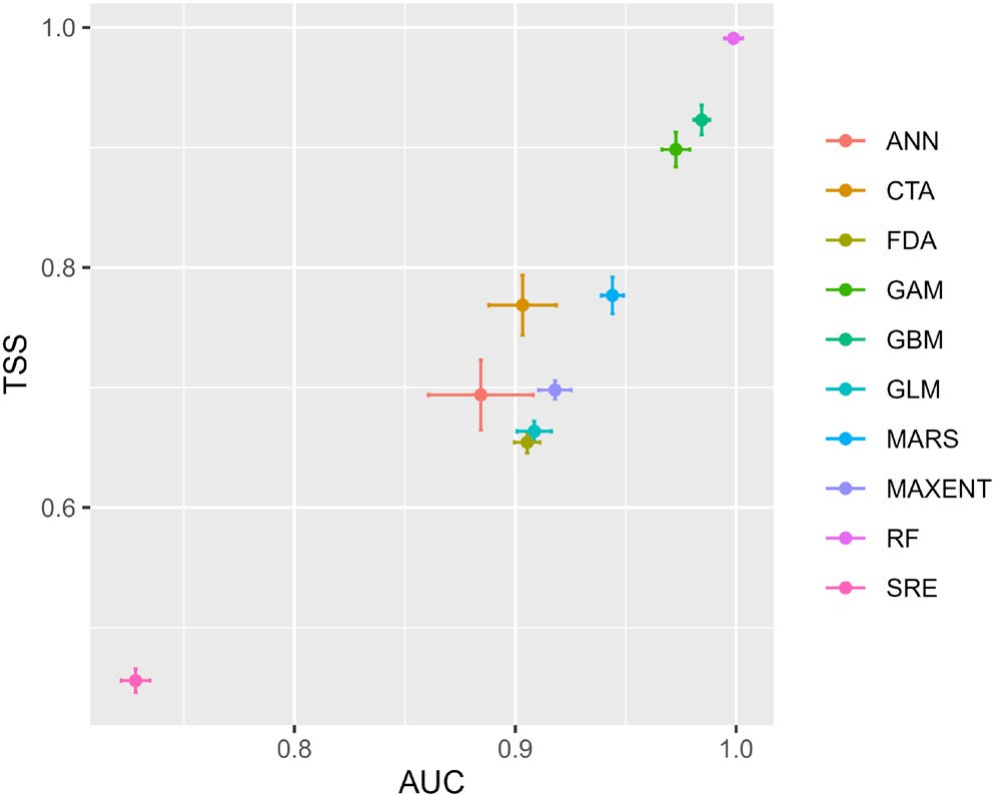
Predictive performance of 10 selected models with their respective AUC and TSS values.

**FIGURE 3:**
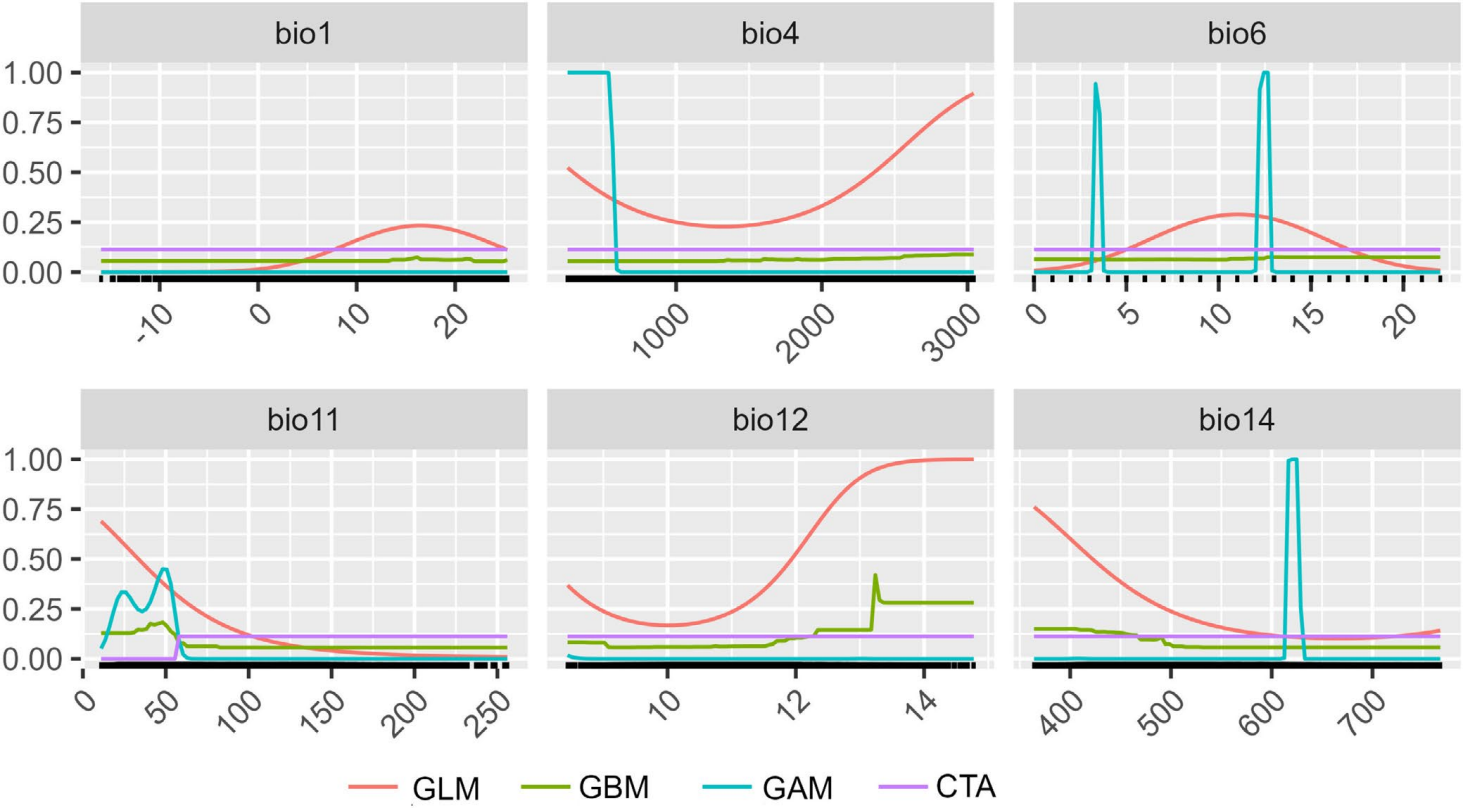
The response curve showing the maximum probability of occurrence of tree‐ferns in different environmental variables: Bio11–mean temperature of coldest quarter, Bio14–precipitation of driest months, Bio1–annual mean temperature, Bio4–temperature seasonality, Bio12–annual precipitation, Bio6–minimum temperature of coldest months in descending order for selected algorithms (GLM, GAM, GBM, CTA).

The predictive ensemble model performance was good, with TSS values 0.45–0.99 and AUC values 0.72–0.99 (Figure 2). The predictive models GAM, GBM, GLM, CTA, MARS, and RF performed better in modeling (TSS value 0.65–0.99 and AUC value 0.89–0.99) than Maxent, ANN, FDA, and SRE (Figure 2). Four algorithms (GAM, GBM, CTA, GLM) with TSS > 0.65, AUC > 0.89, and high accuracy and excellent performance were subjected to correlation analysis and employed for modeling. Among the variables tested, the predictive variables Bio11—mean temperature of the coldest quarter, Bio14—precipitation in driest months, and Bio1—annual mean temperature contributed > 33% each, and the other variables Bio4—temperature seasonality and Bio12—annual precipitation contributed 27% and 17%, respectively, and stood as important for determining the distribution of tree‐ferns (Figure 3).

### 3.2 Population and Distribution

The population density greatly varied at district and site levels, with the highest density (207/ha) in Kaski district (Table 1). We recorded a total of 275 individuals of tree‐ferns from 65 quadrats from 11 districts of Nepal (Table 1) within the altitudinal range of 300–2500 m (Figure 1). The distribution record of tree‐ferns was reported from 28 districts, including 11 districts of Table 1. The other 17 districts with the occurrence records of tree‐ferns were Bhojpur, Dhankuta, Jhapa, Morang, and Sankhuwasawa from Koshi province; Chitwan, Dhading, Dolakha, Lalitpur, and Makwanpur from Bagmati province; Gorkha, Manang, Parbat, and Syangja from Gandaki province; and Palpa, Rolpa, and Rukum from Lumbini province (Figure 1).

**TABLE 1:** Study sites, their altitudinal range and population density data of tree‐ferns in Nepal and supported literature.

Province	District, sites and av. altitudinal range	Density/ha	Earlier reports
Gandaki	Kaski (Pame, Chapakot, Panchase, Maramche, Bhujrung khola, Siding, Polyang, Thakuri Basti) (670–1750 m)	207	Fraser‐Jenkins et al. (2015), Thapa (2017), Bhattarai et al. (2018), Present study 2024
Gandaki	Lamjung (Kuncha bazar, Syamgha, Ram bazar, Kavredanda, Jagat) (800–1460 m)	105	Bhuju and Joshi (2009), Bhattarai and Rajbhandary (2017), Bhattarai et al. (2018), Present study 2024
Gandaki	Tanahun (Risti khola) (630 m)	25	Fraser‐Jenkins et al. (2015), Bhattarai et al. (2018), Present study 2024
Bagmati	Kathmandu (Sankhu, Sundarijal) (1400–1600 m)	102	Fraser‐Jenkins et al. (2015)
Bagmati	Kavre (Dhulikhel, Khawa (1300 m)	60	Dhamala et al. (2021))
Bagmati	Ramechap (Manthali) (760 m)	50	Bhuju and Joshi (2009), Bhattarai and Rajbhandary (2017)
Bagmati	Bhaktapur (Nilbarahi) (1300 m)	12	Fraser‐Jenkins et al. (2015)
Koshi	Ilam (Kutidada, Fikkal Godak, Jasbire, Kharitar, Rungsung, Pashupatinagar (300–1840 m)	105	Bhuju and Joshi (2009), Fraser‐Jenkins et al. (2015))
Koshi	Taplejung (Phungphunge jharana, Saagu, Aase Tribeni Fulbari, Mitlung) (1960–2000 m)	51	Fraser‐Jenkins et al. (2015), Present study 2024
Koshi	Solukhumbu (Phaplu) (1700 m)	10	Fraser‐Jenkins et al. (2015), Present study 2024
Koshi	Terhathum (Hartung khola) (1350 m)	7	Present study 2024

**FIGURE 4:**
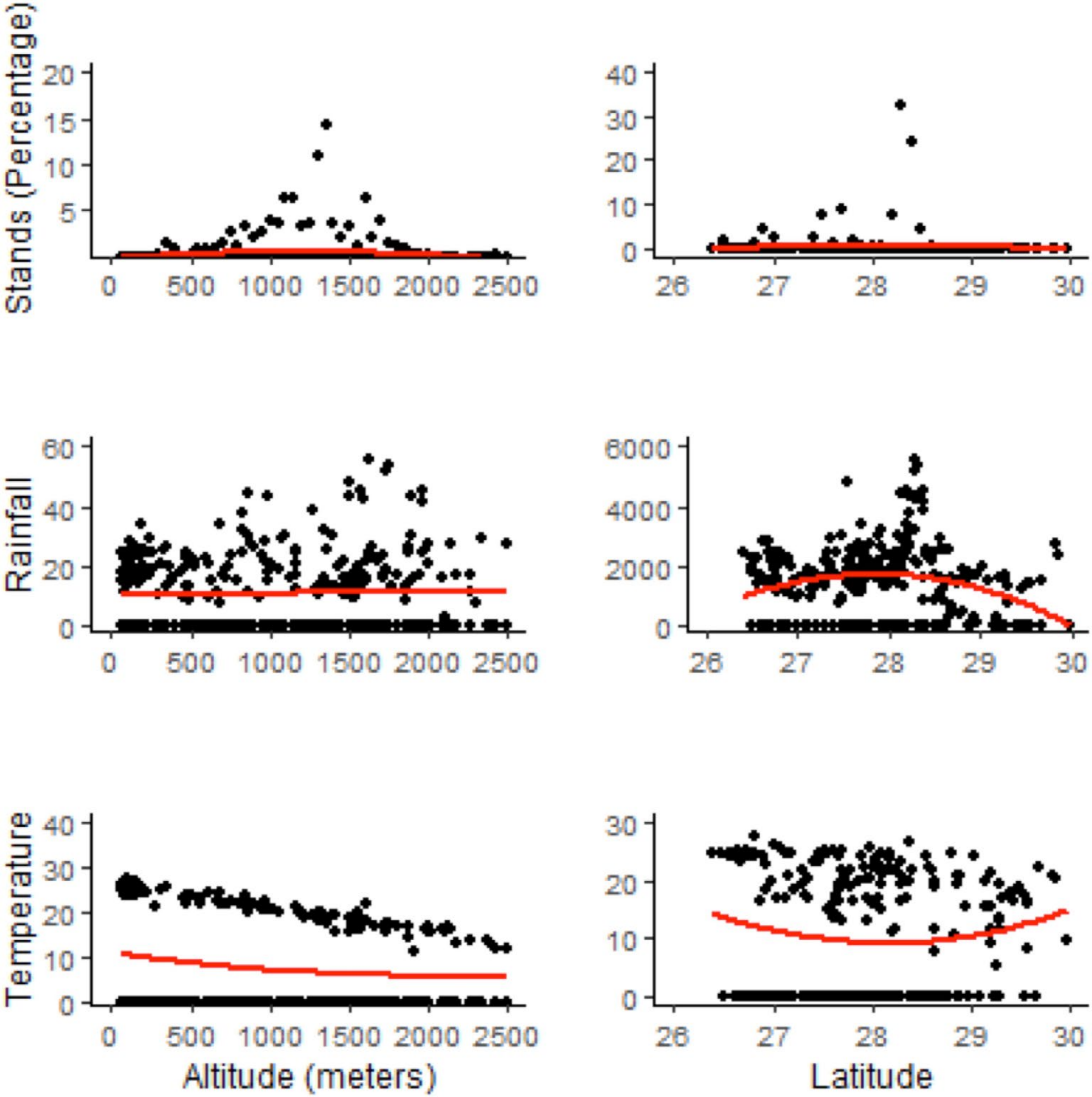
Scatter plots showing the distribution of tree‐ferns (top two), rainfall (middle two) and temperature (bottom two) along the altitudinal and latitudinal gradients.

Tree‐ferns were recorded mainly from the shady and moist areas near the stream and gullies and frequently associated with plant species *Alnus nepalensis, Castanopsis indica, Engelhardtia spicata*, and *Schima wallichii*. As the plants are shade‐loving, there were tree‐ferns found growing at forest fringes, and a good population was observed at the fringes of *the Schima‐Castanopsis* forest of *Birim‐Jaichare‐Dadakharka*, Chapakot, and *Bhujrung* khola habitat of Kaski. Scatter plots of 247 geocoordinates points (Figure 4) showed that the distribution was clumped, observed over 60% of total stands at 1000–1500 m altitude with a significant positive association (*p* = 0.0018) and over two‐thirds at 28°–28.5° N latitude with an insignificant positive association (*p* = 0.74). At the longitude level, 83°–88° E (Central and East Nepal) was the best. There is a negative association between latitude and temperature and latitude and rainfall with *p* = 0.5359 and *p* = 0.1537, respectively (Figure 4).

### 3.3 Potential Distribution

**FIGURE 5:**
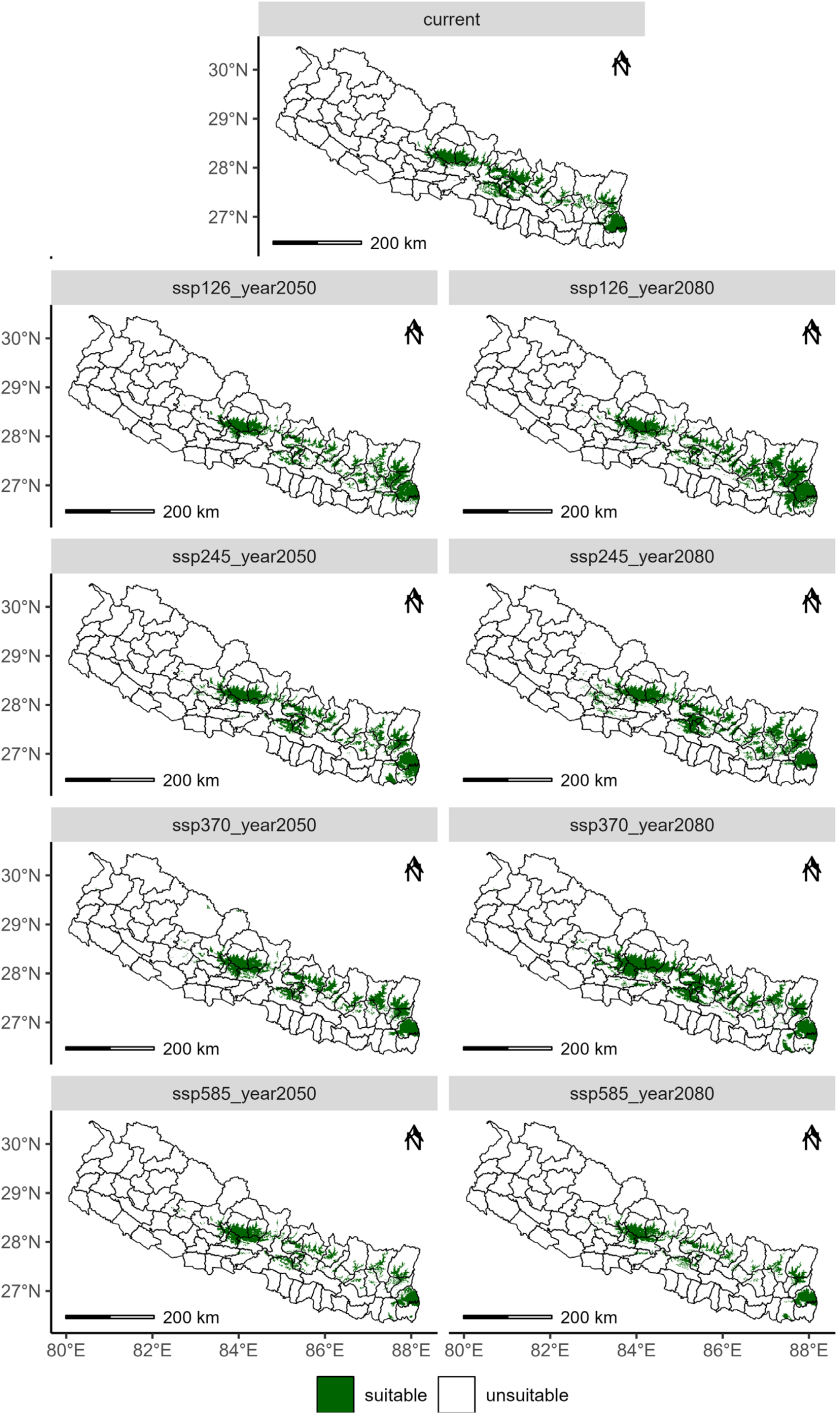
Distribution of tree‐ferns under present and future (2050, 2070, SSP1‐2.6, SSP2‐4.5, SSP3‐7.0 and SSP5‐8.5) bioclimatic conditions categorizing habitat suitability as suitable and unsuitable.

**FIGURE 6:**
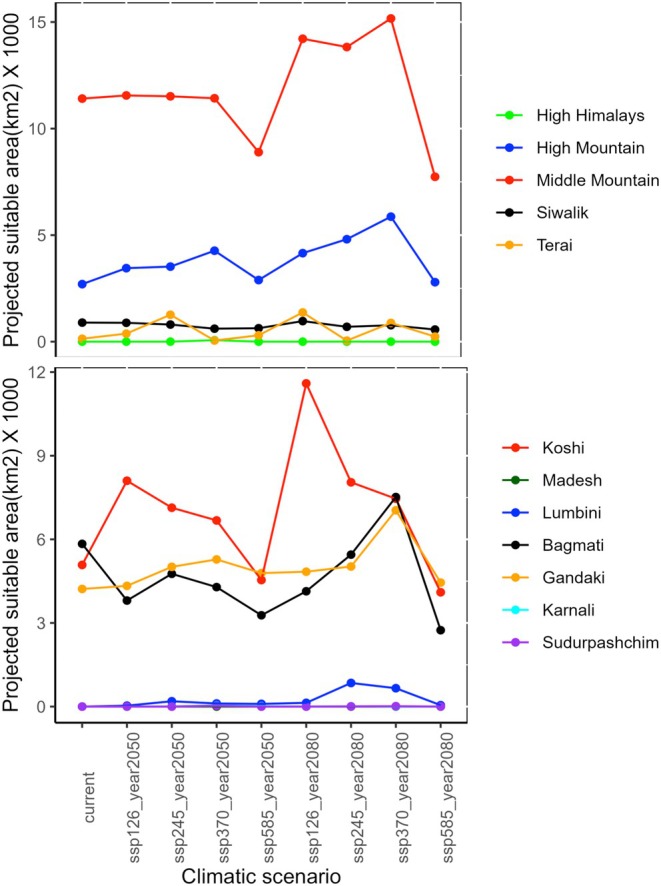
Projected average suitable area of tree‐ferns in each physiographic zone (above) and province (below) under different climatic conditions.

Our results indicate that the average total suitable habitat for tree‐ferns in Nepal is 16864 km2 (11.5% of the total area of Nepal). Over time, the average value of suitable area will expand by 12.8%, yet some contractions are predicted at district level and SSP585 scenario. The area was projected to increase by 3.2% in 2050 and 22% in 2080 at national level; however, the increase will be greatly varied at the level of scenario (SSP126, SSP245, SSP370, SSP585), projection year (2050, 2080), province, physiographic zone, and latitudinal and longitudinal gradients. The sharp decrease will likely happen by −16% in SSP585‐2050 and −25% in SSP585‐2080. Among the scenarios, we chose SSP245 (middle‐of‐the‐road scenario) to interpret the results and discussion. In the SSP245 scenario, the suitable areas for tree‐ferns will likely increase by 12.9% in 2050 and 28% by 2080 (Figures 5 and 6). At the province level, a profound increase of 40% and 58% will be reported in Koshi province, respectively, for 2050 and 2080, followed by 18.7% and 19% in Gandaki province, while the 18% contraction of suitable habitats will occur in Bagmati province in SSP245 scenario.

Although the population and presence points were reported from 28 districts of Gandaki, Koshi, Bagmati, and Lumbini provinces, the modeling shows the distribution is likely in 38 districts of the five provinces (Gandaki, Koshi, Bagmati, Lumbini, and Madhesh), with the highest area in Bagmati province 5837 km^2^. As the modeling shows an expansion of habitats, the future potential distribution may cover 44 districts in 2050 and 49 districts in 2080 with profound distribution in mid‐mountains (Figure 6) within 82.5°–88° E longitude and 26°–29° N latitude. The projected suitable area is greater and is constantly increasing in the selected scenarios in the middle mountains, followed by high mountains (Figure 6). In the Siwalik and Tarai regions, the predicted suitable area was around 2500 km^2^, which was projected to increase insignificantly in 2080. The average values of the present distribution of tree‐ferns in Ilam, Sindhupalchok, Kaski, Lamjung, and Makawanpur districts were the highest, while that in the future will be shrunk in Sindhupalchok, Kavre, Dhading, and Makawanpur districts, all four of which are from Bagmati province.

## 4 Discussion

### 4.1 Modeling and Environmental Variables

Our model's TSS and AUC values were 0.45–0.99 and 0.72–0.99, respectively, indicating that this model is statistically robust and has good predictive performance. Among the 10 models tested in modeling, RF, GAM, and GBM contributed the most (TSS > 0.89 and AUC > 0.95). We found the GAM pertinent in modeling the distribution of threatened plants like tree‐ferns since the GAM result was reported as helpful in finding the potential areas of threatened and endemic medicinal tree plants (Antunez et al. 2017). GAM, the most widely used statistical modeling tool to analyze the relationship between the distribution of the species and their environment, is based on the use of non‐linearity and non‐parametric regression that allows the flexible description of complex species responses to the environment (Leathwick et al. 2006). GAM and GBM showed the highest accuracy while modeling *Dactylorhiza* and *Rheum* medicinal plants (Wani et al. 2022). GAM and GLM (regression model), and GBM were most influential in finding the potential distribution of medicinal plants in Nepal (Kunwar et al. 2023). The maximum TSS value (> 0.8) was obseved in GBM for a sub‐tropical medicinal plant (*Paris polyphylla*) (Kunwar et al. 2021).

Among the tested variables used in this study, precipitation‐related predictive variable Bio14 (precipitation of driest months) and temperature‐related predictive variable Bio11 (mean temperature of the coldest quarter) surpassed other variables as the main factors affecting the future distribution of tree‐ferns. Each contributed the most (> 34.5%). Precipitation is essential in determining the distribution of relict tree‐ferns because they need moisture for their growth and development (Kessler et al. 2011). Increases in precipitation have been shown to enhance species richness and plant diversity markedly by promoting soil moisture variability (Bhatta et al. 2021). Precipitation in the driest months (Bio14) was a significant factor affecting fern growth in the forest (De Gasper et al. 2015).

We compared our result with one carried out earlier (WWF Nepal 2022) (Table 2). The comparative analysis purported that the climate extreme variables (maximum temperature in warmest month—Bio5, minimum temperature in the coldest month—Bio6, precipitation in the wettest month—Bio13, precipitation in the wettest quarter—Bio16) are insignificant in determining the distribution of medicinal plants. The place with a tendency to have precipitation in the driest month (Bio14) and average temperature (Bio1) in a season (temperature seasonality‐Bio4, mean temperature of the coldest quarter‐Bio11) without extremes (isothermality) harbors the moderate distribution and population of tree‐ferns. Precipitation in the driest month supports high plant species richness, which is attributed to the high regeneration success (Gwitira et al. 2014). Thus, the tree‐ferns are impacted by climate change as climate change decreases the precipitation of the driest quarter (De Gasper et al. 2021). The northern parts of Karnali (west of 83° E) with the least population of tree‐ferns received the least precipitation in 2023 (DHM 2023).

**TABLE 2:** Climate change impacts distribution modeling of tree‐ferns in Nepal.

Species	Top five environmental variables played a role in distribution (in descending order from left to right)					Model	Reference
Tree‐ferns	Bio 11	Bio 14	Bio 1	Bio 4	Bio 12	eSDM	Present study, 2024
Tree‐ferns	Bio 18	Bio 3	Bio 9	Bio 19	Bio 2	MaxEnt	WWF Nepal 2022

### 4.2 Population of Tree‐Ferns

The report of 275 individuals from 11 districts and the remnant/planted stands from 17 other districts shows that the population of tree‐ferns in Nepal at present covers 28 districts of central and eastern Nepal with a fragmented and threatened population, supporting that there is no pure forest stand of tree‐ferns. This account adds six districts (East Rukum, Rolpa, Makwanpur, Ramechap, Dhankuta, and Tehrathum) to the earlier records of 22 districts in Nepal (Bhuju and Joshi 2009; Fraser‐Jenkins et al. 2015; Bhattarai and Rajbhandary 2017; Dhamala et al. 2021).

Tree‐ferns density per hectare was 207 in Kaski (see Figure 6), which was higher than the earlier record (204/ha) of WWF Nepal (2022) and less than 225/ha of Bhattarai et al. (2018). The remnant population is fragmented and primarily confined to central (Bhattarai et al. 2018) and eastern Nepal (Bhuju and Joshi 2009) and in the moist areas near the rivers, gorges, streams, and gullies. It is frequently found with *Alnus nepalensis, Daphniphyllum himalayense, Engelhardtia spicata, Macaranga pustulata* as associates and grows luxuriantly under *Schima wallichii*‐*Castanopsis indica* forest. Tree‐ferns prefer moist places (Budha et al. 2023) and riverine habitats (Singh 2020). Hence, tree‐ferns are an indicator species of the moist and humid climate. They generally grow in light gaps in the canopy to avoid the light effects (Wei et al. 2021; Xie et al. 2022). The distribution of tree‐ferns is between 300 m and 2500 m altitude, with the maximum at 1000–1500 m asl and subtropical bioclimate, reported as a natural range of distribution (WWF Nepal 2015, Thapa 2017, Poudel et al. 2018, Dhamala et al. 2021). This area coincides with the areas that receive maximum rainfall. The abundance of tree‐ferns at higher elevations (above 1500 m) depends on the moisture availability (Kluge et al. 2006). Nepal's mid‐hill (1000–3000 m), characterized by a variety of broadleaf and pine forests (DFRS 2015), boasts a good number of tree‐ferns (TISC 2010).

### 4.3 Distribution Modeling

Despite the threatened and fragmented population, the modeling predicted that the suitable area for tree‐ferns would increase by 12.8%, consenting and dissenting from the previous study results of the Himalayas (Shrestha et al. 2022; Wani et al. 2022). The suitable areas of tree‐ferns were projected to increase in the middle and high mountains in different scenarios, which might be related to an increase in soil temperature, which supports the increase of plant species (Verma et al. 2023). This might be due to extending suitable plant habitats in the higher areas (Zhang et al. 2021). On the contrary, tree‐ferns cannot tolerate increasing temperatures in the lower areas (Trethowan et al. 2023). It is evident that tropical areas are facing a high impact of climate change and invasive species (Cahyaningsih et al. 2021); the tree‐ferns endemic to tropical humid riverine areas will face increasing threats induced by both climatic and anthropogenic factors. The current tree‐ferns population and its distribution areas are positively associated (*p* = 0.017), revealing that the population will be augmented in increasing suitable habitats. However, the expansion of suitable areas may not guarantee increased plant production (Rana et al. 2017). Thus, the climate change has mixed effects on the population and distribution of relict plant species including tree‐ferns (Lu et al. 2023).

Among the seven provinces, Koshi and Gandaki provinces will have the most suitable areas for tree‐ferns, followed by Bagmati and Lumbini. This might be related to soil moisture content as Koshi and Gandaki possess more soil moisture index (Talchabhadel et al. 2019), and they support more biodiversity, including tree‐ferns. According to Fraser‐Jenkins et al. (2015), all five species of tree‐ferns are found in eastern Nepal (Koshi province), whereas only three are in central Nepal (Bagmati and Gandaki provinces), while their distribution in west Nepal is yet to be explored. According to Thapa (2002) and Rajbhandary (2016), they are found from east to west Nepal. The projected population increase in Gandaki province (Kaski and Lamjung districts), central Nepal, could be related to high soil moisture content attributed to the high rainfall in the province (Thakur and Rajbhandary 2018). The central southern slope of the Himalayas receives the maximum rainfall (Dahal 2012), resulting in lush habitats for plant biodiversity (FoN 2024). Central mountains are projected as the future medicinal plant hub (Shrestha et al. 2022; Kunwar et al. 2023).

Tree‐ferns are distributed null in far western Nepal and maximum in central and eastern Nepal, with the densest population in Kaski and Lamjung districts. This reveals that central mid‐hills (1000–3000 m asl) with moist habitats are appropriate for tree‐ferns. Gurung (1991) mentioned that tree‐ferns are distributed in central and eastern Nepal up to 2250 m altitude (Stainton 1988). The west‐to‐east increase in suitable habitats of tree‐ferns is correlated with the increased intensity of the monsoon in the east, supported by the finding of Lilleso et al. (2005). The distribution record of tree‐ferns in Manang, Nepal (Shrestha and Rajbhandary 2019; Poudel et al. 2018) and that from China (Dong 2009; Yuan et al. 2023) and from Uttarakhand to Sikkim, India (Kholia et al. 2013) shows that it has a potential extended range of distribution across trans‐Himalaya and throughout Nepal.

### 4.4 Conservation

Uses of tree‐ferns as food, medicine, and ornament are common in Nepal (Adhikari et al. 2019; Kandel 2020; Ojha and Devkota 2021; Dhamala et al. 2021; Bhattarai and Kunwar 2022), revealing that the species may get overharvested in the near future in concurrence with food unavailability. Overexploitation of plant parts, compounded with climate change, grazing, and degradation, has resulted in a tree‐fern population and distribution threatened. Tree‐ferns are declining rapidly in the wild (Dixit and Singh 2004). However, their national‐level conservation status has not been assessed so far in Nepal. The species conservation plan of the tree‐ferns is urged for its evolutionary and ethnobotanical significance. Our research identified suitable habitats for tree‐ferns by constructing an ensemble model to predict the potential distribution. Thus, the effects of climate change on the population and distribution of tree‐ferns should be further scaled up to improve our understanding of the dynamics of people, plants, and climate change interactions.

## 5 Conclusions

The modeling showed that the suitable area for tree‐ferns will be extended in the future, yet there are some contractions predicted at the level of province, district, and climate scenarios. Tree‐ferns at present are dominant in Central and Eastern Nepal, with maximum density in the Kaski and Lamjung districts, revealing that central mid‐hills with moist habitats, maximum rainfall, and moderate disturbances boast more population and habitats for tree‐ferns at the current period. Moreover, the future distribution will concentrate in the middle mountains of the Koshi and Gandaki provinces, revealing a close association of tree‐ferns, soil moisture, and precipitation. However, the tree‐ferns, a relict tree species endemic to tropical riverine areas, will face increasing threats induced by climatic and anthropogenic disturbances. Climate extreme variables are constraining the future distribution of tree‐ferns, whereas temperature seasonality (Bio4), precipitation in the driest month (Bio14), and mean temperature of the coldest quarter (Bio11) support the population and distribution. It is evident that tropical areas face a high impact of climate change and plant invasion; the tree‐ferns, endemic to tropical humid riverine areas, will face increasing threats, drawing immediate conservation measures. We recommend devising a tree‐ferns conservation action plan emphasizing participatory and climate‐resilient conservation actions and utilizing this study's findings as a baseline for understanding the current and future distribution scenario.

## 6 Limitations

The rarefaction of data in 1 km^2^ resulted in less precise potential distribution, which could be one limitation of this study. The Shared Socio‐economic Pathways (SSP126, SSP245, SSP370, SSP585) employed in this study are based on certain assumptions and predictions. Future climate change and socio‐economic development may differ from these scenarios. This means that the prediction results presented may deviate from the actual situation and cannot accurately reflect the distribution changes of tree‐ferns in the real future environment. Moreover, the prediction of future distribution may be overestimated because the SSP modeling is based on assumptions and projections about future conditions rather than on direct observations. This modeling is based on the georeference points authors collected from the sample districts and available in the secondary sources. Since the convenience sampling was followed, some of the inaccessible and remote sites where the tree‐ferns could probably have grown might have been missed in this modeling. Thus, the present modeling result may not accurately reflect the prediction results on a national scale. However, it is noted that even with some limitations, this study contributes to a growing body of literature that argues for the application of SSP modeling in assessing the impact of climate change on the future distribution of species.

## Author Contributions

**Ripu M. Kunwar:** conceptualization (equal), data curation (equal), funding acquisition (equal), methodology (equal), writing – original draft (equal), writing – review and editing (equal). **Dipak Khadka:** data curation (equal), methodology (equal), writing – original draft (equal), writing – review and editing (equal). **Khum Thapa‐Magar:** formal analysis (equal), methodology (equal), writing – review and editing (equal). **Binaya Adhikari:** data curation (equal), formal analysis (equal), writing – review and editing (equal). **Durga H. Kutal:** formal analysis (equal), methodology (equal), writing – review and editing (equal). **Rama Ghimire:** data curation (equal), formal analysis (equal), methodology (equal), writing – review and editing (equal). **Komal R. Kafle:** funding acquisition (equal), methodology (equal), resources (equal), writing – review and editing (equal). **Sony Baral:** data curation (equal), formal analysis (equal), resources (equal), writing – review and editing (equal). **Gokarna J. Thapa:** funding acquisition (equal), methodology (equal), resources (equal), writing – review and editing (equal). **Ananta Bhandari:** funding acquisition (equal), resources (equal), writing – review and editing (equal).

## Conflicts of Interest

The authors declare no conflicts of interest.

## Supporting information


**File S1.** The result of climate change modeling with different scenario at province and district level.

**File S2.** Field geo‐coordinate points of tree‐ferns in Nepal.

## Data Availability

The data supporting this study's findings are provided as a supplemental file. The spatial distribution data of tree‐ferns were from online sources, including Flora of Nepal (https://www. floraofnepal.org), iNaturalist (https://www.inaturalist.org/), the Global Biodiversity Information Facility (https://www.gbif.org/), the Royal Botanic Garden at Edinburgh, United Kingdom (RBGE; https://data.rbge.org.uk/search/herbarium/), and the Herbarium at the University of Tokyo, Japan (TI; https://umdb.um.u‐tokyo.ac.jp/DShokubu/). Additional points were collected from the voucher specimen records of the National Herbarium and Plant Laboratories (KATH) https://plantdatabase.kath.gov.np/plants/search, Tribhuvan University Central Herbarium (TUCH), and literature (Bhuju and Joshi 2009, Thapa 2017, Bhattarai et al. 2018, Poudel et al. 2018, Dhamala et al. 2021 and WWF Nepal 2022).
